# Special Issue “Advanced Research on Immune Cells and Cytokines (2nd Edition)”

**DOI:** 10.3390/ijms262311416

**Published:** 2025-11-26

**Authors:** Athanasia Mouzaki

**Affiliations:** Laboratory of Immunohematology, Department of Internal Medicine, Medical School, University of Patras, 26504 Patras, Greece; mouzaki@upatras.gr

## 1. Introduction

Cytokines and immune cells are fundamental to intercellular communication within the immune system, orchestrating processes essential for defense, repair, and immune tolerance. Rapid progress in molecular profiling, bioinformatics, and imaging technologies has expanded our understanding of cytokine–cell interactions and their implications for disease mechanisms and therapeutic intervention. Building on the success of the first edition, this Second Edition of the Special Issue “Advanced Research on Immune Cells and Cytokines” presents original research and reviews that deepen our understanding of immune cell dynamics and cytokine biology across a wide range of pathologies.

The studies presented in this Special Issue advance our understanding of immune cell heterogeneity and cytokine-driven signaling in inflammation, infection, and malignancy. Collectively, they show that immune cell function is shaped by a complex interplay of environmental cues, molecular pathways, and disease context. The integration of high-throughput profiling, in vivo modeling, and translational approaches highlighted in these works underscores the progress toward precision immunology and cytokine-targeted interventions.

We expect that the insights provided here will inspire further research into cytokine biology, immune modulation, and cross-disciplinary strategies for diagnosis and therapy. Continued exploration of cytokine networks promises to reveal new therapeutic opportunities in immune-mediated and degenerative diseases.

The Guest Editor gratefully acknowledges all contributing authors for their high-quality submissions, the expert reviewers for their insightful evaluations, and the *IJMS* editorial team for their professional support throughout the publication process.

## 2. Highlights of Research Contributions

### 2.1. Cytokine Regulation in Inflammatory Skin Diseases

Three contributions explore inflammation within cutaneous immune environments.

Windorfer et al. (contribution 1) used multi-antigen analysis to profile dendritic cell subsets in chronic eczema and psoriasis, identifying a unique CD163/CD63^+^ monocyte-derived DC subset that distinguishes eczema from psoriasis and reflects disease-specific immune signatures.

Knoch et al. (contribution 2) examined gene expression and immune cell composition across lichen planus subtypes, uncovering marked transcriptional heterogeneity and immune pathway activation patterns that support subtype-specific therapeutic strategies.

Toledano-Macías et al. (contribution 3) demonstrated that radiofrequency current (CRET) stimulation modulates keratinocyte cytokine production through the EGFR/ERK1/2/NF-κB pathway, highlighting an innovative, non-pharmacological approach to regulating inflammation.

### 2.2. Cytokines in Infectious and Systemic Disorders

Cytokine-mediated immune modulation plays pivotal roles in infection and systemic inflammation.

Gao et al. (contribution 4) compared macrophage-derived factors between HIV-1 and HIV-2 infections, identifying differential regulation of CXCL7 and consistent M-CSF induction as determinants of viral replication and pathogenicity.

Naidoo and Naicker (contribution 5) reviewed the dualistic role of interleukin-10 (IL-10) in HIV infection comorbid with preeclampsia, delineating its paradoxical effects on immune tolerance and viral persistence, and emphasizing its clinical relevance in pregnancy-related immune regulation.

### 2.3. Immunomodulation and Environmental Stress

Gao et al. (contribution 6) established a hypobaric hypoxia-induced immune imbalance model in mice and showed that Astragaloside IV mitigates spleen pathology by restoring Th1/Th2 cytokine equilibrium and reducing oxidative stress. These findings provide valuable insight into the therapeutic potential of natural compounds targeting cytokine pathways in stress-induced immune dysregulation.

### 2.4. Cytokine Networks in Cancer and Neurodegeneration

Maggisano et al. (contribution 7) provided a comprehensive overview of the IL-20 cytokine subfamily (IL-19, IL-20, IL-22, IL-24, and IL-26) and their dual functions in cancer biology—ranging from tumor-promoting to tumor-suppressive effects—via modulation of macrophage polarization and the tumor microenvironment.

Goldansaz et al. (contribution 8) demonstrated that lipopolysaccharide and recombinant prion protein can independently or synergistically induce neurodegenerative changes in mice, even without classical PrP^Sc^ accumulation, illustrating how inflammation intersects with protein misfolding in prion-like pathologies.

### 2.5. Chemokine Landscapes Linking Inflammation and Cancer

Zhang et al. (contribution 9) presented an integrative transcriptomic analysis of chemokine expression across inflammatory bowel disease and colorectal cancer, identifying four distinct chemokine expression clusters associated with disease progression. Their work provides a comprehensive atlas of chemokine regulation from inflammation to carcinogenesis and serves as a resource for therapeutic discovery.

## 3. Outlook

An enormous amount of data has been generated by recent advances in biotechnology and bioinformatics, helping us better understand the “big picture” of immune cells and cytokines. Cytokines orchestrate immune cell behavior across tissues, and recent single-cell and spatial omics have sharpened our view of this dialogue. Large compendia now map cell type-specific transcriptional responses to dozens of cytokines, revealing both canonical and unexpected targets, while spatial frameworks and new computational tools resolve cytokine-driven niches in situ. Together, these datasets show that cytokine signaling is highly context dependent, varying by tissue, microenvironment, and cell state [1,2,3].

Innate-adaptive crosstalk has been redefined by studies on innate lymphoid cells (ILCs), which are plastic sentinels at barrier sites and shape downstream T-cell responses. Tissue cues condition ILC function, and updated models emphasize how local niches imprint ILC phenotypes. In parallel, focused studies at mucosal surfaces highlight synergistic cytokine circuits—exemplified by IL-17 and IL-22 cooperation in barrier defense—and the specialized roles of Th22 cells in intestinal homeostasis [4,5,6,7].

Innate immune “memory” (trained immunity) adds a temporal layer to cytokine biology. Epigenetic and metabolic reprogramming of myeloid cells retunes cytokine production and responsiveness, with implications spanning infection, autoimmunity, cancer, and cardiovascular disease. Emerging translational efforts are exploring small molecules and vaccine strategies to modulate this training axis [8,9,10,11,12].

Dysregulated cytokine networks remain central to pathology. Refined mechanistic accounts of cytokine storm and cytokine release syndrome link feed-forward inflammatory loops to clinical deterioration, while disease-focused syntheses (e.g., in inflammatory bowel disease) integrate innate and adaptive cytokine sources to guide targeted therapies [13,14,15,16].

Therapeutically, precision delivery and metabolic context are frontiers. CD45-targeted immunocytokines illustrate strategies to concentrate cytokine activity on desired cell subsets and tissues, potentially widening the therapeutic window. Meanwhile, immunometabolic reviews underscore how amino acid pathways such as arginine and arginase govern cytokine programs and effector functions [17,18].
